# Rapid *in vitro* detection of CTX-M groups 1, 2, 8, 9 resistance genes by LAMP assays

**DOI:** 10.1371/journal.pone.0200421

**Published:** 2018-07-18

**Authors:** Odile Lalainasoa Rivoarilala, Benoît Garin, Felamboahangy Andriamahery, Jean Marc Collard

**Affiliations:** 1 Unité Bactériologie Expérimentale, Institut Pasteur de Madagascar, Madagascar; 2 Département de Sciences de la vie et de l’Environnement, Faculté des Sciences, Université d’Antananarivo, Madagascar; Seconda Universita degli Studi di Napoli, ITALY

## Abstract

**Background:**

The prevalence of bacteria producing CTX-M Extended-Spectrum β-lactamases (ESBLs) has increased around the world and some of them became a major cause of infections such as bloodstream or urinary tract infections (UTI). We developed a loop-mediated isothermal amplification (LAMP) assay for a simple, rapid and sensitive detection of the four most common CTX-M groups, namely CTX-M groups 1, 2, 8 and 9.

**Methods:**

LAMP primers targeting the four ESBLs CTX-M groups were designed using the Primer Explorer V4 software. The detection limit of the method was tested by serial dilution of reference DNAs. The primer specificity of the LAMP reaction was tested on DNA extracted from six strains producing various group of CTX-M and validated using DNA extracted from CTX-M-resistant clinical isolates (isolated from pus, urine, or blood). Results were compared with those of conventional PCR.

**Results:**

We were able to detect down to 0.1 pg/ul of DNA using the newly developed LAMP assays whereas the minimal amount detectable for conventional PCR was 50 to 100pg/ul, indicating that the LAMP assay was found to have a detection limit at least 500 to 1000 times lower than the PCR. Additionally, representative genes from the CTX-M groups 1, 2, 8 and 9 were amplified using the designed assay and no cross amplification was observed between the four CTX-M groups, demonstrating the specificity of the LAMP assay. Of the 37 clinical strains tested, the four LAMP assays showed 100% sensitivity and 87%, 97%, 100%, 100% specificity for the CTX-M groups 1, 2, 8 and 9 respectively.

**Conclusion:**

Being sensitive, specific, rapid and standard methods, the LAMP assays developed in this study have a potential to be beneficial tools in molecular epidemiology and surveillance studies of the four prevalent EBSLs CTX-M groups even in low cost laboratory.

## Introduction

The overuse and oftentimes the misuse of β-lactams antibiotics are the most important factors promoting the selection and emergence of β-lactam-resistant bacteria in both human and veterinary medicine [1]. The first β-lactamases, TEM-1, TEM-2 and SHV-1 were found in *Escherichia coli* and *Klebsiella pneumoniae* strains and described in the early 1960s, about 15 years after the wide use of penicillin. Few years later, these β-lactamases spread worldwide and were found in different species of Enterobacteriaceae. TEM-1, TEM-2 and SHV-1 were named narrow-spectrum β-lactamases due to their ability to hydrolyze penicillins and the first generation of cephalosporins, such as cephalothin, cephaloridine or cefazolin [2]. Twenty years later, novel β-lactam antibiotics, in particular extended-spectrum cephalosporins were developed to mitigate the emergence of these narrow-spectrum β-lactamases enzymes. Nevertheless, the selection pressure due to the overuse of extended-spectrum cephalosporins has selected for new variants of β-lactamases. These enzymes are known as extended-spectrum β-lactamases (ESBLs), because of their increased spectrum of activity, especially against 2nd-, 3rd- and 4th-generation cephalosporins and monobactams (e.g. aztreonam), besides penicillins. ESBLs-producing bacteria remain however sensitive to carbapenems, cephamycins (e.g. cefoxitin), and β-Lactamase inhibitors such as clavulanic acid [3,4]. ESBLs are usually plasmid mediated which facilitate their transfer between bacteria. Nowadays, more than 600 ESBL have been described (http://www.lahey.org/Studies/), the majority of which are members of either the CTX-M (for **c**efo**t**a**x**imase) families and TEM-1/2, SHV-1 β-lactamases mutants. The TEM, SHV and CTX-M-types β-lactamases belong to a fairly heterogeneous lineage of molecular class A active site-serine β-lactamases. CTX-M-type β-lactamases can be further differentiated into at least six sub-lineages or groups, namely CTX-M-1, CTX-M-2, CTX-M-8, CTX-M-9, CTX-M-25, and KLUC [5].

ESBLs are among the most significant resistance determinants spreading worldwide mainly in *E*. *coli* and especially in *E*. *coli* ST-131 [6] and *K*. *pneumoniae* which became a major cause of infections such as urinary tract infection (UTI) or bloodstream infections [7–9]. Several studies have confirmed the widespread of CTX-M-type β-lactamases in many countries, mainly CTX-M-14 (CTX-M group 9) and CTX-M-15 (CTX-M group 1) enzymes [10].

Diagnosis of ESBL resistance is recommended for patient treatment and necessary for surveillance. Routine methods for ESBLs detection include ESBL E-tests and the double-disk synergy test (DDST) [11]. These methods are culture-based assays and therefore require several days and experienced staff. Attempts to detect these bacterial resistances by genotypic approaches are currently in development such as amplification by polymerase chain reaction (PCR). However, Taq DNA polymerase in PCR assays can be readily inactivated by inhibitors present in crude biological samples [12]. Moreover, PCR requires expensive equipment (thermal cyler) and subsequent electrophoresis to visualize amplicons or fluorochromes for the real-time PCR, what limits its use in the field.

Loop-mediated isothermal amplification (LAMP), a simple, rapid and sensitive method of DNA amplification was developed by Notomi *et al*. in 2000 [13]. This method is based on an auto-cycling strand displacement DNA synthesis performed by the *Bst* DNA polymerase and the use of four to six specific primers (two outer primers F3 and B3, two inner primers FIP and BIP, two loop primers LF and LB) [13–15]. The amplification by LAMP is implemented under isothermal conditions ranging from 60°C to 65°C for about 60min requiring only a simple water bath or a heat block. LAMP-positive products result in large amounts of amplification target DNA which facilitate their observation with the naked eyes by turbidity or by adding fluorescent intercalating agents such as Sybr green dye [16,17] or metal ion indicators such as hydroxynaphthol blue [18]. Therefore, LAMP method has been classified as a powerful tool which facilitates rapid detection of bacteria, virus and parasites [19] and will be a reliable and low cost clinical diagnosis method.

The aim of this study was to establish simple, rapid and sensitive detection assays based on LAMP technology for the four most common CTX-M groups, namely CTX-M groups 1, 2, 8 and 9.

## Materials and methods

### Bacterial strains and clinical samples

β-lactamase producing strains used in this study have been provided by the National Reference Center (NRC) for antibiotic resistance, France and presented in Table 1.

**Table 1:** Bacterial strains used for the specificity of LAMP assay in this study.

Strain reference	Bacterial species	β-lactamases	Gene sequences
Concord 09–3534	*Salmonella* sp.	CTX-M group 1	*bla*_CTX-M-3_, *bla*_CTX-M-15_
U2A 1790	*E*. *coli*	CTX-M group 1	*bla*_CTX-M-28_
NC185cro	*Salmonella* sp.	CTX-M group 2	*bla*_CTX-M-2_
U2A 2145	*Salmonella* sp.	CTX-M group 2	*bla*_CTX-M-4_
U2A 2251	*Klyuvera georgiana*	CTX-M group 8	*bla*_CTX-M-8_
U2A 1796	*E*. *coli*	CTX-M group 9	*bla*_CTX-M-9_

### Preparation of DNA templates

For bacterial DNA extraction, a few single colonies from a plate were suspended in 200μl of distilled water, placed into a boiling water bath for 10 min, subsequently transferred on ice for 5 min and then centrifuged at 12 000 rpm/min for 5 min. Five microliter (5μl) of the supernatant were used as DNA template in each reaction.

### LAMP primer design

Nucleotide sequences in the four groups of CTX-M β-lactamase genes were selected from the National Center for Biotechnology information (NCBI). The accession numbers of these sequences were X92506.1, Y10278.1, AF255298.1, AY005110.1, AF305837.1, AY044436.1, AY080894.1, AF488377.1 for the CTX-M group 1; AB770487.1, Y14156, U95364, AJ005044, AJ005045.1, AJ416344.1 for the CTX-M group 2; AF189721.1 for the CTX-M group 8; AF174129, AF252623, AF252622, AY029068, AY033516, AJ416346.1, AY143430.1, AY156923.1 for the CTX-M group 9. These sequences were aligned using BioEdit 7.2.5 software [20]. Consensus sequences were analysed with the LAMP primers design software “Primer Explorer V4” software (http://primerexplorer.jp/e/) and specific primers were automatically designed. The four basic primers used for LAMP were a forward inner primer (FIP), a backward inner primer (BIP) and two outer primers (F3 and B3) [14]. One or two Loop primers (LF and LB) were designed for each target. These last are optional but can be used to accelerate the reaction time [21]. Primers were validated using BLAST software (http://www.ncbi.nlm.gov/BLAST) to ensure their specificity. The list of used primers is shown in Table 2.

**Table 2:** LAMP primer sets used in this study.

Primers	Sequence (5’ to 3’)
**CTX-M group 1**	
**F3**	CACTGCGTCAGTTCACGC
**B3**	CACGGCCATCACTTTACTGG
**FIP**	TTGCTGTACGTCCGCCGTTTGTTTTCAACCGTCACGCTGTTGT
**BIP**	CAGTCGGGAGGAAGACTGGGTTTTTGCGCTCATCAGCACGATA
**LF**	TACAGCGGCACACTTCCTA
**CTX-M group 2**	
**F3**	GGTGGTCCCGATAAAGTGAC
**B3**	CTGTGCCCGCTGAGTTTC
**FIP**	CGCCTGGAATGGCGGTATTGAGTTTTGGGTGATGAGACCTTCCGT
**BIP**	GTGATACCACCACGCCGCTCGCTTTACCCAGCGTCAGAT
**CTX-M group 8**	
**F3**	ATTAGCGATGGCGCAGAC
**B3**	CCAGATAACGGCGATGTCAT
**FIP**	AGCCACGTTACCAGTTGCGCTTTTCAATCTGACGTTGGGCAGT
**BIP**	CTGCCAGCATTCAGGCTGGGTTTTGTCGTACCATAATCACCGCT
**LF**	CGCTGAGTTTCACCTAAGGC
**CTX-M group 9**	
**F3**	TGCGCTGGGCGAAACC
**B3**	GGCTCTCTGCGTTCTGTTG
**FIP**	GGTAAGCCGGCCCGAATGCTTTTTGTTGGTGACGTGGCTCAA
**BIP**	GGCACCACCAATGATATTGCGGCGGCTGGGTAAAATAGGTCA
**LF**	CGCCGGTCGTATTGCCT
**LB**	TGATCTGGCCGCAGGGT

### Optimization of LAMP reactions

The first LAMP assays were performed with the conditions described in the original paper of Notomi *et al*. [14] and subsequently optimized to get the best amplification results with the designed primers. The reaction was carried out in a 25μl mixture containing 0.2μM of each outer primers F3 and B3, 0.8μM of each inner primers FIP and BIP, and when available 0.4μM of each loop primers LF or/and LB, 20mM Tris-HCl (pH 8.8), 10mM KCl, 10mM (NH4)_2_SO_4_, 0.1% Tween 20, 1M betaine, 4mM MgSO_4_, 0.4mM each deoxynucleotide triphosphate, 8U *Bst* DNA polymerase and 5μl of DNA extract. Distilled water (DW) was used as negative control. The reaction was carried out in an Eppendorf tube and incubated at 65°C in a thermo block or in a water bath. To optimize this reaction, optimal condition for factors affecting the efficiency of LAMP amplification were examined. The parameters tested were: betaine concentration (0.6–1.2M), MgSO_4_ concentration (4 – 8mM), reaction time (30 – 60min) and temperature (59–67°C). Two different methods were used to detect LAMP products. For direct visual inspection, 1μl of Sybr green I 10 000X (http://www.sigmaaldrich.com) diluted 1/10 was added to 25μl of the reaction mixture. A positive reaction is indicated by a color change from orange to yellow, while a negative reaction has no color change. The visible color change could be observed by naked eyes. As an additional proof, products from the reaction were also analyzed by gel electrophoresis in which 5μl of the LAMP product were loaded on a 2% agarose gel.

### Specificity of LAMP assay

To evaluate the specificity of LAMP primer sets, DNA extracts from strains producing various β-lactamases (Table 1) were used in a LAMP assay under optimal conditions. Subsequently, CTX-M group 1 and CTX-M group 2 LAMP products were digested by the *Hha*I restriction enzyme, and CTX-M group 8 and CTX-M group 9 LAMP products by *Bbv*I. The digestion products were analyzed by electrophoresis on a 2% agarose gel. These experiments were performed at least in duplicate to ensure repeatability.

### Comparison of detection limit of conventional PCR and LAMP assays

To compare LAMP and PCR amplification assays in terms of sensitivity (limit of detection), genomic DNA was serially diluted 10-fold in sterile distilled water, ranging from 1 to 10^−7^ng/μl of DNA. LAMP was performed under optimal condition as previously described. For conventional PCR amplification, the assay was performed with primers listed in Table 3. The amplification was carried out in a 25μl total reaction volume with 2μl genomic DNA as template, 1X PCR Solys Biodyne Buffer (20mMTris-HCl, 10mMKCl, 2mM MgSO_4_, pH 8.8), 0.6M of betaine, 0.5μl (10μM) of a couple of appropriate primers (CTX-M group-F/CTX-M group-R), 0.5μl of dNTPs mixture (2.5mM of each dNTPs), and 0.125μl (5U/ml) of Taq DNA polymerase (Solis Biodyne). The thermal cycle profile for PCR was 1 initial cycle at 95°C for 4 min, followed by 30 cycles (95°C for 40 sec, the annealing temperature of each group of CTX-M primers (Table 3) for 1 min and 68°C for 1 min of extension) and a final extension at 68°C for 5 min. The PCR products were then analyzed on a 1.5% agarose gel by electrophoresis, and the amplicons were visualized by staining with ethidium bromide.

**Table 3:** Sequences of oligonucleotides used in conventional PCR.

Primer	Primer sequence (5’– 3’)	Position	Annealing temp*(°C)	Product (pb)	Reference
**CTX-M group 1**	(+) ATGGTTAAAAAATCACTGCG	63–82	61	861	[22]
	(-) TTGGTGACGATTTTAGCCGC	928–909			
**CTX-M group 2**	(+) ATGATGACTCAGAGCATTCG	6–25	56	861	[22]
	(-) TGGGTTACGATTTTCGCCGC	871–852			
**CTX-M group 8**	(+) GCGGCGCTGGAGAAAAGCAG	123–142	61	608	[22]
	(-) GCTGCCGGTTTTATCCCGA	712–731			
**CTX-M group 9**	(+) ATGGTGACAAAGAGAGTGCA	1–20	57	870	[22]
	(-) CCCTTCGGCGATGATTCTC	851–870			

*temp: temperature

### Antimicrobial susceptibility testing

Clinical Enterobacteriaceae isolates (isolated from pus, urine, or blood) used in this study were obtained from the Clinical Center of Biology, Pasteur Institute of Madagascar, Antananarivo. Antimicrobial susceptibilities of the isolates were determined by the disk diffusion method on Mueller-Hinton agar (Bio-Rad, France), as recommended by the Antibiogram Committee of the French Microbiology Society (ACFMS 2015).

### ESBL confirmation by the double-disk synergy method

The presence of ESBLs in clinical isolates was confirmed by the double-disk synergy (DDST) test. DDST was performed by placing the disk of cefotaxime (30 μg), ceftazidime (30 μg), and combination of amoxicillin/clavulanic acid (20 μg/10 μg) on a lawn culture of bacteria on Muller-Hinton agar plate, with a 20 mm distance between each disk center to center. Then, plates were incubated at 37°C for 18–24 hrs. Enhancement of the inhibition zone between the disks containing clavulanic acid and cefotaxime or ceftazidime indicated the presence of ESBL production [23]. *E*. *coli* ATCC 25922 and *K*. *pneumoniae* ATCC 700603 were used as internal quality control strains.

## Results

### Optimization of LAMP reaction

To optimize LAMP conditions, DNA extracted from *E*. *coli* U2A 1790, *Salmonella* spp. U2A 2145, *Salmonella* spp. U2A 2251, *E*. *coli* U2A 1796 carrying the group 1, 2, 8 and 9 of CTX-M gene respectively were used as a template. Results showed that the optimal conditions for the LAMP amplification reaction of each CTX-M group were 0.8M betaine, 7mM MgSO_4_ (data not shown).

The use of loop primers are not the essential requirement but its presences can reduce LAMP reaction time [21]. Generated loop primers could be only one, LF or LB, as it was the case for primers of CTX-M groups 1 and 8, or both primers LF and LB at the same time, as it was the case of primers CTX-M group 9 (Table 2). In the presence of these loop primers, LAMP reaction were detected positive from 45 min of amplification. For CTX-M group 2 LAMP assay, we observed that the presence of these primers resulted false-positive results, which required systemic assessment and optimization of the reaction (data not shown). Indeed CTX-M2 LAMP assay without loop primers was established and the positivity time of the reaction was 60 min (data not shown). Thereby the standard reaction time for LAMP amplification in this study was set at 60 min and the temperature was at 63°C. These conditions provided the best result for the four LAMP amplifications both by eye visualization and on agarose gel (data not shown). These tests were repeated two to five times and results were identical for each.

### Detection limit of LAMP and PCR assays

**Fig 1:**
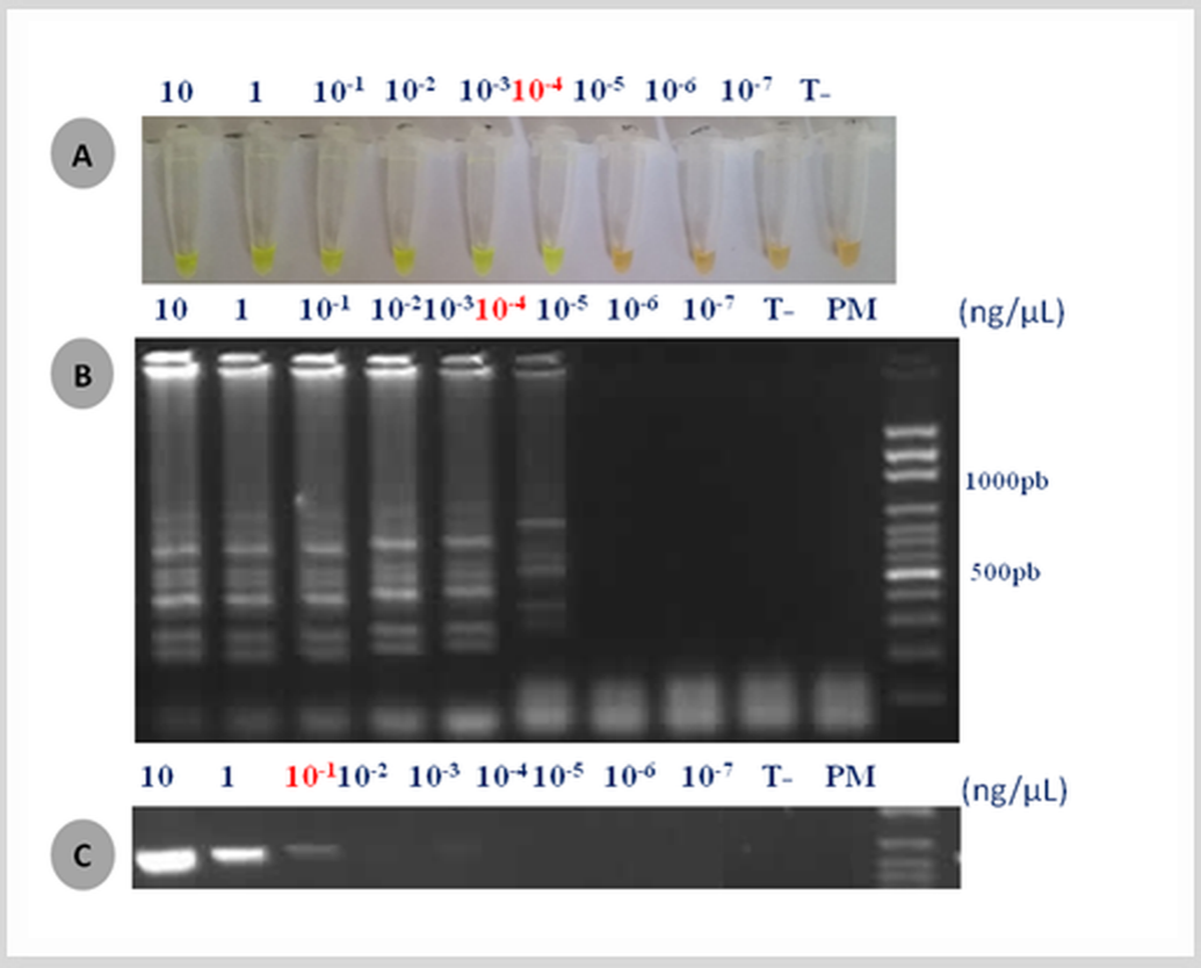
Detection limits of the loop-mediated isothermal amplification (LAMP) and PCR for detection of CTX-M group 1. Various amount of template DNA (10, 1, 10^−1^, 10^−2^, 10^−3^, 10^−4^, 10^−5^, 10^−6^, 10^-7^ng/μl) were used: (A) eye visualization of the LAMP reaction after coloration with the Sybr Green I dye, (B) visualization after migration on an agarose gel of the LAMP products. (C) Visualization after migration on an agarose gel of conventional PCR products.

As shown in Fig 1A and 1B with CTX-M group 1 as example, the detection limit of LAMP for the four CTX-M groups was 100fg/μL. Detection limit of PCR CTX-M group 1 and 2 was 100pg/μL (Fig 1C) while this was 50 to 100pg/μl for CTX-M group 8 and 9 (data not shown). These results showed that the detection limit of LAMP assays conducted in our study were around 500 to 1000 fold lower than conventional PCR assays. These tests were repeated three to five times and results were the same.

### Specificity of LAMP assay

**Fig 2:**
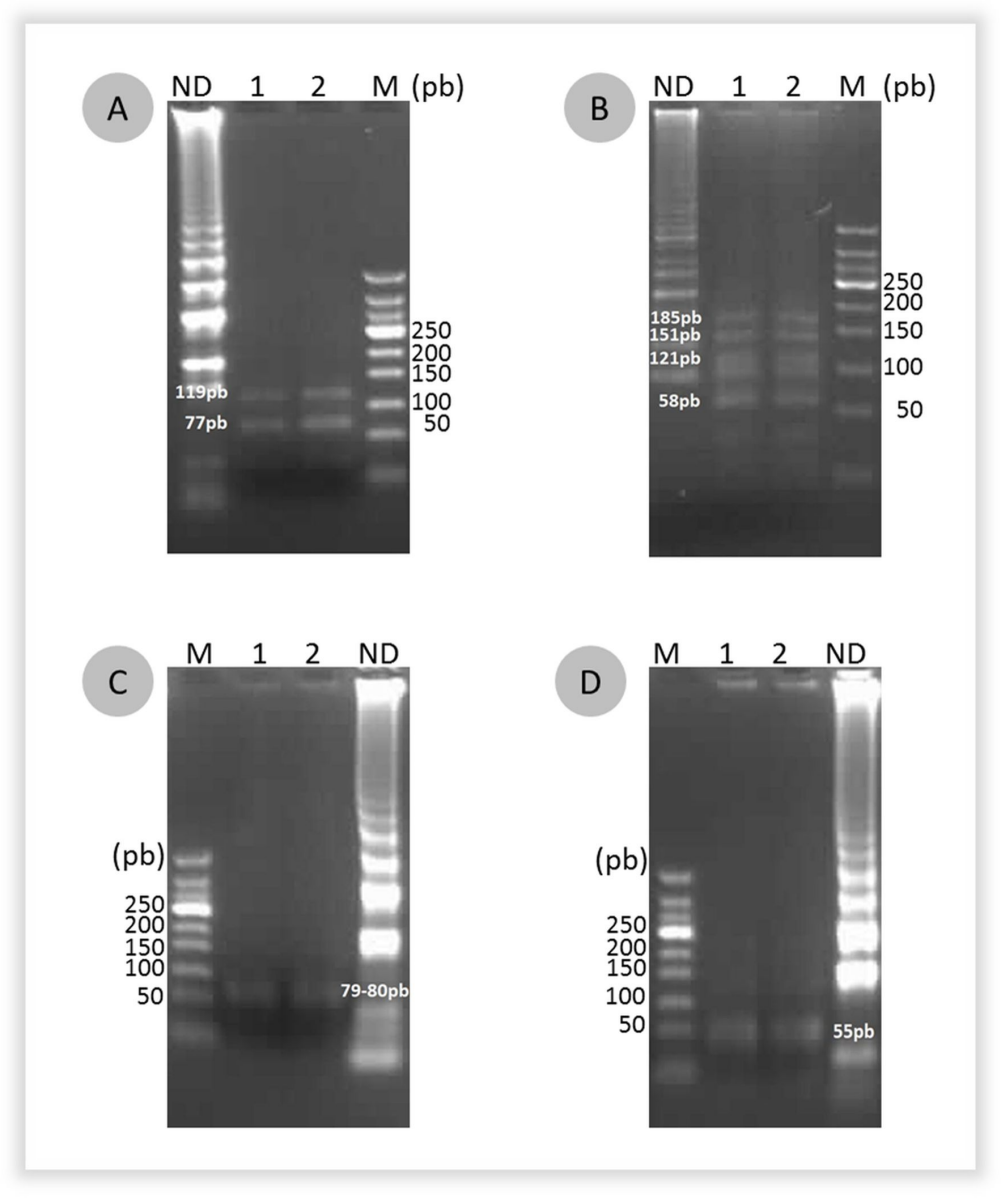
Restriction analysis of LAMP products for the four CTX-M groups. (A) *Hha*I digestion of CTX-M group 1, (B) *Hha*I digestion of CTX-M group 2, (C), *Bbv*I digestion of CTX-M group 8, (D) *Bbv*I digestion of CTX-M group 9. Lane 1: positive LAMP product undigested, lane 2 and 3: digestion product of positive LAMP, M: DNA ladder marker 50pb.

Six various β-lactamase resistance genes (Table 1) were tested to evaluate the specificity of the designed LAMP primers. Positive LAMP amplification was observed with the specific genes encoding β-lactamases, no cross amplification were observed between the four different CTX-M groups. The LAMP results were consistent with those obtained for conventional PCR amplifications. Furthermore, digestion of LAMP positive products by restriction enzymes resulted into DNA fragments with the predicted sizes. Digestion of LAMP products amplified by CTX-M groups 1 and 2 primers by *Hha*I resulted in respectively 2 bands (77 – 119bp) and 3 bands (121-151-185bp) while the digestion of CTX-M group 8 and group 9 by *Bbv*I produced respectively (79 – 80pb) and 55pb. These results indicate the high specificity of our LAMP assays (Fig 2).

### Application of LAMP assays on clinical isolates

Thirty seven clinical isolates belonging to the Enterobacteriaceae family and producing an ESBL confirmed by DDST were included in this study. The optimized LAMP reactions for the four CTX-M groups were evaluated by using DNA from boiled bacteria of these clinical isolates. Of the 37 strains tested, 30 (81%) were positive for the presence of CTX-M group 1 based on LAMP assay, while 29 (78%) strains were found positive by PCR method considered as the gold standard. For the detection of CTX-M group 2, two strains (2/37, 5%) were detected positive by LAMP while only one strain (1/37, 3%) was detected by PCR. For CTX-M group 8 and group 9, three (3/37, 8%) for each group were found positive by both PCR and LAMP (Table 4). One strain was positive for CTX-M group 1 and group 9 by both LAMP and PCR assays. Specificity, sensitivity, Positive Predictive Value (PPV) and Negative Predictive Value (NPV) were showed in Table 4.

**Table 4:** Detection of CTX-M group 1, 2, 8 and 9 in clinical isolates by LAMP assays and PCR amplification.

LAMP assay	PCR assay		Sensitivity % [IC]	Specificity % [IC]	PPV^a^ % [IC]	NPV^b^ % [IC]
	Positive	Negative				
**CTX-M group 1**						
Positive	29	1	100 [88–100]	87 [47–99]	96 [82–99]	100
Negative	0	7				
**CTX-M group 2**						
Positive	1	1	100 [2–100]	97 [85–99]	50 [12–87]	100
Negative	0	35				
**CTX-M group 8**						
Positive	3	0	100 [29–100]	100 [89–100]	100	100
Negative	0	34				
**CTX-M group 9**						
Positive	3	0	100 [29–100]	100 [89–100]	100	100
Negative	0	34				

LAMP: loop mediated isothermal amplification

^a^: Positive Predictive value

^b^: Negative Predictive value

## Discussion

Until the end of 1990s, most of the ESBLs detected were of mutants of SHV and TEM types. From 2000, ESBLs CTX-M-types were detected and become a major problem of global magnitude. Most of the enzymes within this family confer resistance to third generation cephalosporins [24]. CTX-M group 1, specifically *bla*_CTX-M15_, has been reported to be the most prevalent genotype among ESBL producing isolates followed by *bla*_CTX-M14_ from the CTX-M group 9 which has been frequently reported in some regions of Europe and Asia [5][25,26]. The prevalence of these ESBLs were particularly high in some enterobacterial species (e.g., *K*. *pneumoniae* and *E*. *coli*) [3][27,28]. The CTX-M groups 2 and 8 have been reported in Asia, particularly in *Salmonella* species [29]. CTX-M group 25 β-lactamases has been observed less frequently worldwide. Subsequently this group was not developed in this study.

The routine diagnostic currently used to detect CTX-M resistant organisms include antibiotic susceptibility testing, double disk synergy test (DDST) or the use of automated instruments for determining the minimum inhibitory concentration of antibiotics. Although easy to perform, these culture-based routine diagnostic methods require 16-20h to obtain a result and sometimes require additional confirmation tests. Moreover, they do not provide the specific type of ESBL. The commonly used molecular diagnostic method for detecting CTX-M resistant organisms is PCR amplification. This method is used for its ability and velocity to detect specific target genes. However, PCR method requires expensive equipment restricting its use in routine laboratories, mainly in low-income countries [30].

Novel LAMP assays for the rapid detection of CTX-M group 1, CTX-M group 2, CTX-M group 8, and CTX-M group 9 with a high specificity and a low detection limit were carried out in this study.

The LAMP detection assay was successfully performed within 60min at 63°C. Including DNA extraction step, the assay can be completed within 90min. The LAMP method is less time consuming than conventional PCR assays which require a minimum of 2–4h to a detectable result. The LAMP reaction was carried out at a constant temperature (obtained by a controlled water bath or a heat block) whereas PCR must be performed with a thermal cycler. Moreover, LAMP products could be observed by naked eyes without the need of an electrophoresis [31–33].

Our results showed that the detection limit of CTX-M LAMP assays were around 500 to 1000-fold lower than conventional PCR. These results are in agreement with previous reports [34,35]. The lower detection limit of LAMP assay is due to the use of the four basic primers strengthened to the use of loop primers [21]. The study of Amornrat T., *et al*. showed that the limit of detection of LAMP *bla*_CTX-M-1_ gene was increased 10 fold compared with no loop primers [36]. Otherwise, as used in LAMP test, nested PCR involves the use of two sets of primers. Due to the added couple of primers, this last showed a higher detection limit compared with conventional PCR. In the study of Khan *et al*., LAMP assay was 10 times more sensitive than nested PCR and 100 times sensitive than conventional PCR for the detection of Ypt1 gene [35]. However nested PCR was not tested in this study. Although the use of additional primers such as loop primers (LF or and LB) can enhance the sensitivity and accelerate the LAMP reaction time (< 30min) [21], this sometimes involves false positive results [37,38]. In this study, the unstable reactions particularly false positive results of LAMP CTX-M group 2 due to the presence of loop primers are consistent with what has been found in these previous studies.

Our LAMP assays were also conducted using various CTX-M resistance genes. The assays were shown highly specific with no cross-reactivity between the four CTX-M groups tested. These results demonstrated the highly efficient detection and strong specificity of the LAMP assays developed in our study. Moreover, LAMP products were further digested by restriction enzymes confirming the specificity of the assays.

LAMP and PCR assays were also conducted on 37 ESBL-producers obtained from clinical samples and the detection rates for LAMP CTX-M group 1 (30/37, 81%) and LAMP CTX-M group 2 (2/37, 5%) appear to be similar to those for PCR CTX-M group 1 (29/37, 78%) and group 2 (2/37, 5%). The results showed the high sensitivity (100%) and NPV (100%) for these two LAMP assays. The lower specificity 87%, 97% and PPV 95%, 50% for LAMP CTX-M group 1 and CTX-M group 2 respectively were due to a single discrepancy between the methods. This decrease of specificity may be due to the higher sensitivity of LAMP assay compared to that of conventional PCR assay used as gold standard [39].

However false positive results of LAMP may be caused by the binding of dye to primer dimers or non-specific products, this case of false positive products was not observed in previous studies [36,40,41].

Detection results of CTX-M group 8 and group 9, showed 100% concordance between PCR and LAMP. These data demonstrated the high specificity of the designed LAMP primers for the four CTX-M groups (1, 2, 8, and 9).

On the other side, LAMP has been proven to be a robust method. Kaneko *et al*. have reported that this assay is more tolerant towards inhibitors than conventional PCR [42]. In a previous study, LAMP assays were used to detect the most common carbapenemases and extended-spectrum β-lactamases (ESBLs) in Gram-negative bacteria (GNB) directly from positive blood culture [40]. The LAMP reaction was carried out directly on DNA extracted from blood sample. Another study assessed the simplicity of LAMP in detecting target DNA of *Leptospira* spp. directly from boiled urine or urine pellet samples, without DNA purification step [43].

For the detection of CTX-M group 1 and 9, the amplification time of the LAMP assay developed in this study was a longer (45min) than that of Sergio et *al*., and Zboromyrska et *al*., (15min). However, as a perspective, a single reaction in multiplex of these LAMP CTX-M groups is feasible. Thereby, considerable time and effort can be saved by simultaneously amplifying and detecting two or more target sequences. Although, the differentiation of the origin or specificity of the amplified LAMP products from multiple targets is still challengeable to date, several methods tried to resolve this difficulty such as used real-time detection through annealing curve analysis [44], visual detection by addition of fluorescence-labeled probes [45], colorimetric distinction using an immunochromatographic strip [46].

In conclusion, the four LAMP assays developed in the present study have demonstrated reliable capacities to detect and distinguish four CTX-M different groups. These LAMP assays are sensitive (500 to 1000 times more sensitive than conventional PCR assay), fast (about 60min) and specific to detect CTX-M group 1, 2, 8 and 9 in Enterobacteriaceae isolates. Devices, consumables and reagents are not expensive and may be implemented in laboratories in low income countries. Although a larger number of clinical samples could be studied to ascertain its efficiency, LAMP tests are beneficial tools for use in patient diagnosis and epidemiology surveillance.

## Supporting information

S1 Fig Optimization of betaine concentration for LAMP CTX-M group 1 assay. Various betaine concentrations (0.6M, 0.8M, 1M and 1.2M) were used in the LAMP reaction. MgSO_4_ concentration was maintained at 7mM and the reaction was performed at 65°C– 1H. The reaction products were loaded onto a 2% agarose gel for analysis.
(JPG)

S2 Fig Optimization of betaine concentration for LAMP CTX-M group 2 assay. Various betaine concentrations (0.6M, 0.8M, 1M and 1.2M) were used in the LAMP reaction. MgSO_4_ concentration was maintained at 7mM and the reaction was performed at 65°C– 1H. The reaction products were loaded onto a 2% agarose gel for analysis.
(JPG)

S3 Fig Optimization of betaine concentration for LAMP CTX-M group 8 assay. Various betaine concentrations (0.6M, 0.8M, 1M and 1.2M) were used in the LAMP reaction. MgSO_4_ concentration was maintained at 7mM and the reaction was performed at 65°C– 1H. The reaction products were loaded onto a 2% agarose gel for analysis.
(JPG)

S4 Fig Optimization of betaine concentration for LAMP CTX-M group 9 assay. Various betaine concentrations (0.6M, 0.8M, 1M and 1.2M) were used in the LAMP reaction. MgSO_4_ concentration was maintained at 7mM and the reaction was performed at 65°C– 1H. The reaction products were loaded onto a 2% agarose gel for analysis.
(JPG)

S5 Fig Optimization of MgSO_4_ concentration for LAMP CTX-M group 1 assay. 4mM and 6mM MgSO_4_ concentrations were used in the LAMP reaction. Betaine concentration was maintained at 0.8M and the reaction was performed at 65°C– 1H. The reaction products were loaded onto a 2% agarose gel for analysis.
(JPG)

S6 Fig Optimization of MgSO_4_ concentration for LAMP CTX-M group 1 assay. 7mM and 8mM MgSO_4_ concentrations were used in the LAMP reaction. Betaine concentration was maintained at 0.8M and the reaction was performed at 65°C– 1H. The reaction products were loaded onto a 2% agarose gel for analysis.
(JPG)

S7 Fig Optimization of MgSO_4_ concentration for LAMP CTX-M group 9 assay. Various MgSO_4_ concentrations (4mM, 6mM, 7mM and 8mM) were used in the LAMP reaction. Betaine concentration was maintained at 0.8M and the reaction was performed at 65°C– 1H. The reaction products were loaded onto a 2% agarose gel for analysis.
(JPG)

S8 Fig Determination of the optimal reaction temperature for LAMP CTX-M group 1 assay. With 0.8M of betaine, MgSO_4_ at 7mM and amplification for 1H, the LAMP reaction was performed at 59, 61, 63, 65 and 67°C. The reaction products were visualized after coloration with the Sybr Green I dye.
(JPG)

S9 Fig Determination of the optimal reaction temperature for LAMP CTX-M group 1 assay. With 0.8M of betaine, MgSO_4_ at 7mM and amplification for 1H, the LAMP reaction was performed at 59, 61, 63, 65 and 67°C. The products were loaded onto a 2% agarose gel for analysis.
(JPG)

S10 Fig Determination of the optimal reaction temperature for LAMP CTX-M group 2 assay. Betaine at 0.8M, MgSO_4_ at 7mM and amplification for 1H, the LAMP reaction was performed at 59, 61, 63, 65 and 67°C. The reaction products were visualized after coloration with the Sybr Green I dye.
(JPG)

S11 Fig Determination of the optimal reaction temperature for LAMP CTX-M group 2 assay. With 0.8M of betaine, MgSO_4_ at 7mM and amplification for 1H, the LAMP reaction was performed at 59, 61, 63, 65 and 67°C. The products were loaded onto a 2% agarose gel for analysis.
(JPG)

S12 Fig Determination of the optimal reaction temperature for LAMP CTX-M group 8 assay. With 0.8M of betaine, MgSO_4_ at 7mM and amplification for 1H, the LAMP reaction was performed at 59, 61, 63, 65 and 67°C. The reaction products were visualized after coloration with the Sybr Green I dye.
(JPG)

S13 Fig Determination of the optimal reaction temperature for LAMP CTX-M group 8 assay. With 0.8M of betaine, MgSO_4_ at 7mM and amplification for 1H, the LAMP reaction was performed at 59, 61, 63, 65 and 67°C. The products were loaded onto a 2% gel for analysis.
(JPG)

S14 Fig Determination of the optimal reaction temperature for LAMP CTX-M group 9 assay. Betaine concentration maintained at 0.8M, MgSO_4_ at 7mM and amplification for 1H, the LAMP reaction was performed at 59, 61, 63, 65 and 67°C. The reaction products were visualized after coloration with the Sybr Green I dye.
(JPG)

S15 Fig Determination of the optimal reaction temperature for LAMP CTX-M group 9 assay. With 0.8M of betaine, MgSO_4_ at 7mM and amplification for 1H, the LAMP reaction was performed at 59, 61, 63, 65 and 67°C. The products were loaded onto a 2% gel for analysis.
(JPG)

S16 Fig Determination of the optimal reaction time of LAMP CTX-M group 2 assay. Betaine concentration maintained at 0.8M, MgSO_4_ at 7mM, amplification temperature at 63°C for 1H, the LAMP reaction was performed during 30, 45 and 60min. The reaction products were loaded onto a 2% agarose gel for analysis.
(JPG)

S17 Fig Detection limit of PCR CTX-M group 8 assay. Various amount of template DNA (1, 0.1, 0.05 and 0.01ng/μl) were used. Conventional PCR products were analyzed on agarose gel.
(JPG)

S18 Fig Detection limit of PCR CTX-M group 9 assay. Various amount of template DNA (1, 0.1, 0.05 and 0.01ng/μl) were used. Conventional PCR products were analyzed on agarose gel.
(JPG)

## Abbreviations

ESBLs: Extended-spectrum β-lactamases
SHV: Sulphydrylvariable
TEM: Temoniera
CTX-M: Cefotaximase
LAMP: Loop mediated isothermal amplification
DDST: double disk synergy test
PCR: Polymerase chain reaction
FIP: Forward inner primer
BIP: Backward inner primer
