# Supplementary figures and images for: Rapid in vitro detection of CTX-M groups 1, 2, 8, 9 resistance genes by LAMP assays
Source: PLoS One. 2018 Jul 18;13(7):e0200421. doi: 10.1371/journal.pone.0200421 (PMC6051616; doi:10.1371/journal.pone.0200421)

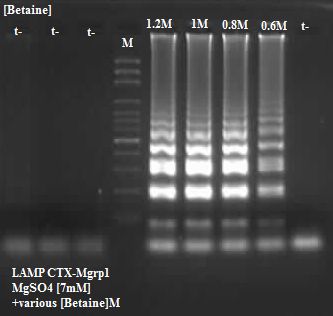

Supplement: S1 Fig — Various betaine concentrations (0.6M, 0.8M, 1M and 1.2M) were used in the LAMP reaction. MgSO4 concentration was maintained at 7mM and the reaction was performed at 65°C– 1H. The reaction products were loaded onto a 2% agarose gel for analysis. (JPG) [file pone.0200421.s001.jpg]

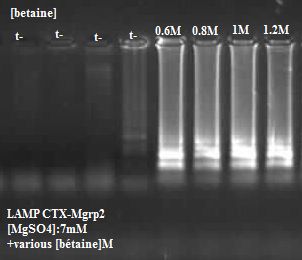

Supplement: S2 Fig — Various betaine concentrations (0.6M, 0.8M, 1M and 1.2M) were used in the LAMP reaction. MgSO4 concentration was maintained at 7mM and the reaction was performed at 65°C– 1H. The reaction products were loaded onto a 2% agarose gel for analysis. (JPG) [file pone.0200421.s002.jpg]

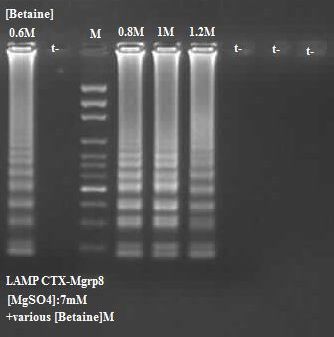

Supplement: S3 Fig — Various betaine concentrations (0.6M, 0.8M, 1M and 1.2M) were used in the LAMP reaction. MgSO4 concentration was maintained at 7mM and the reaction was performed at 65°C– 1H. The reaction products were loaded onto a 2% agarose gel for analysis. (JPG) [file pone.0200421.s003.jpg]

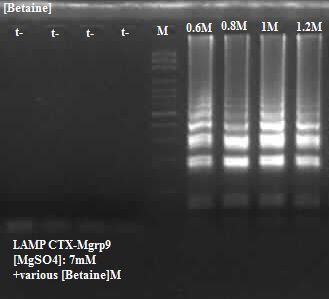

Supplement: S4 Fig — Various betaine concentrations (0.6M, 0.8M, 1M and 1.2M) were used in the LAMP reaction. MgSO4 concentration was maintained at 7mM and the reaction was performed at 65°C– 1H. The reaction products were loaded onto a 2% agarose gel for analysis. (JPG) [file pone.0200421.s004.jpg]

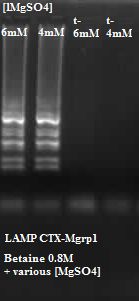

Supplement: S5 Fig — 4mM and 6mM MgSO4 concentrations were used in the LAMP reaction. Betaine concentration was maintained at 0.8M and the reaction was performed at 65°C– 1H. The reaction products were loaded onto a 2% agarose gel for analysis. (JPG) [file pone.0200421.s005.jpg]

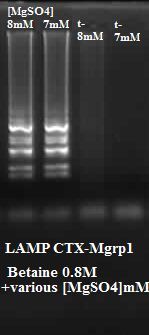

Supplement: S6 Fig — 7mM and 8mM MgSO4 concentrations were used in the LAMP reaction. Betaine concentration was maintained at 0.8M and the reaction was performed at 65°C– 1H. The reaction products were loaded onto a 2% agarose gel for analysis. (JPG) [file pone.0200421.s006.jpg]

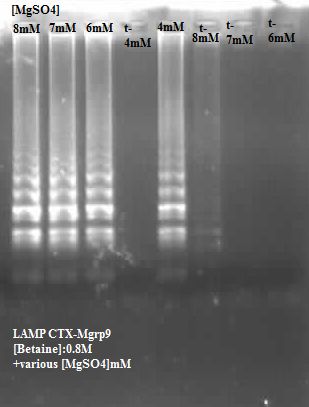

Supplement: S7 Fig — Various MgSO4 concentrations (4mM, 6mM, 7mM and 8mM) were used in the LAMP reaction. Betaine concentration was maintained at 0.8M and the reaction was performed at 65°C– 1H. The reaction products were loaded onto a 2% agarose gel for analysis. (JPG) [file pone.0200421.s007.jpg]

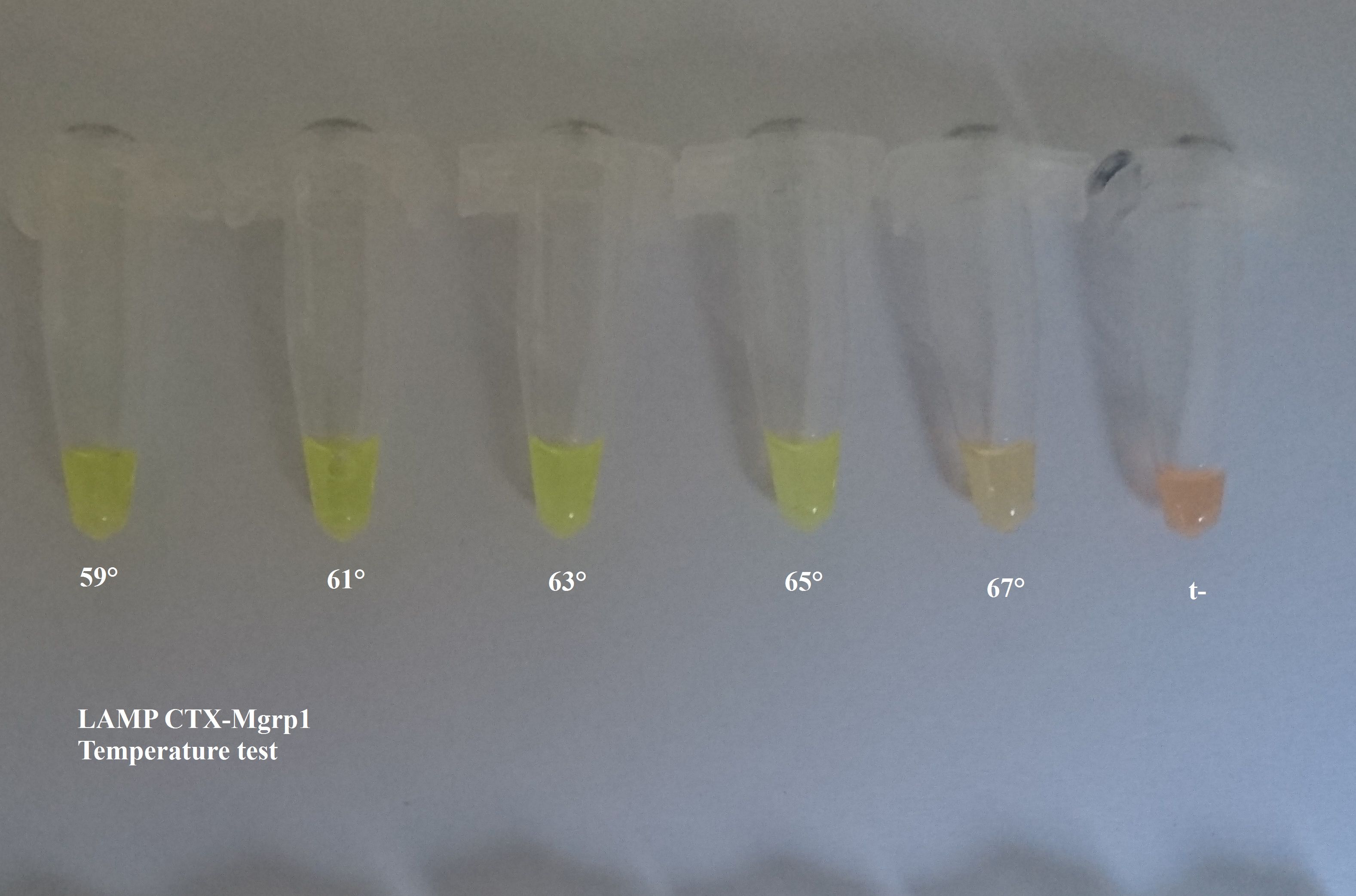

Supplement: S8 Fig — With 0.8M of betaine, MgSO4 at 7mM and amplification for 1H, the LAMP reaction was performed at 59, 61, 63, 65 and 67°C. The reaction products were visualized after coloration with the Sybr Green I dye. (JPG) [file pone.0200421.s008.jpg]

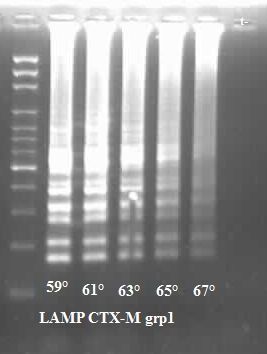

Supplement: S9 Fig — With 0.8M of betaine, MgSO4 at 7mM and amplification for 1H, the LAMP reaction was performed at 59, 61, 63, 65 and 67°C. The products were loaded onto a 2% agarose gel for analysis. (JPG) [file pone.0200421.s009.jpg]

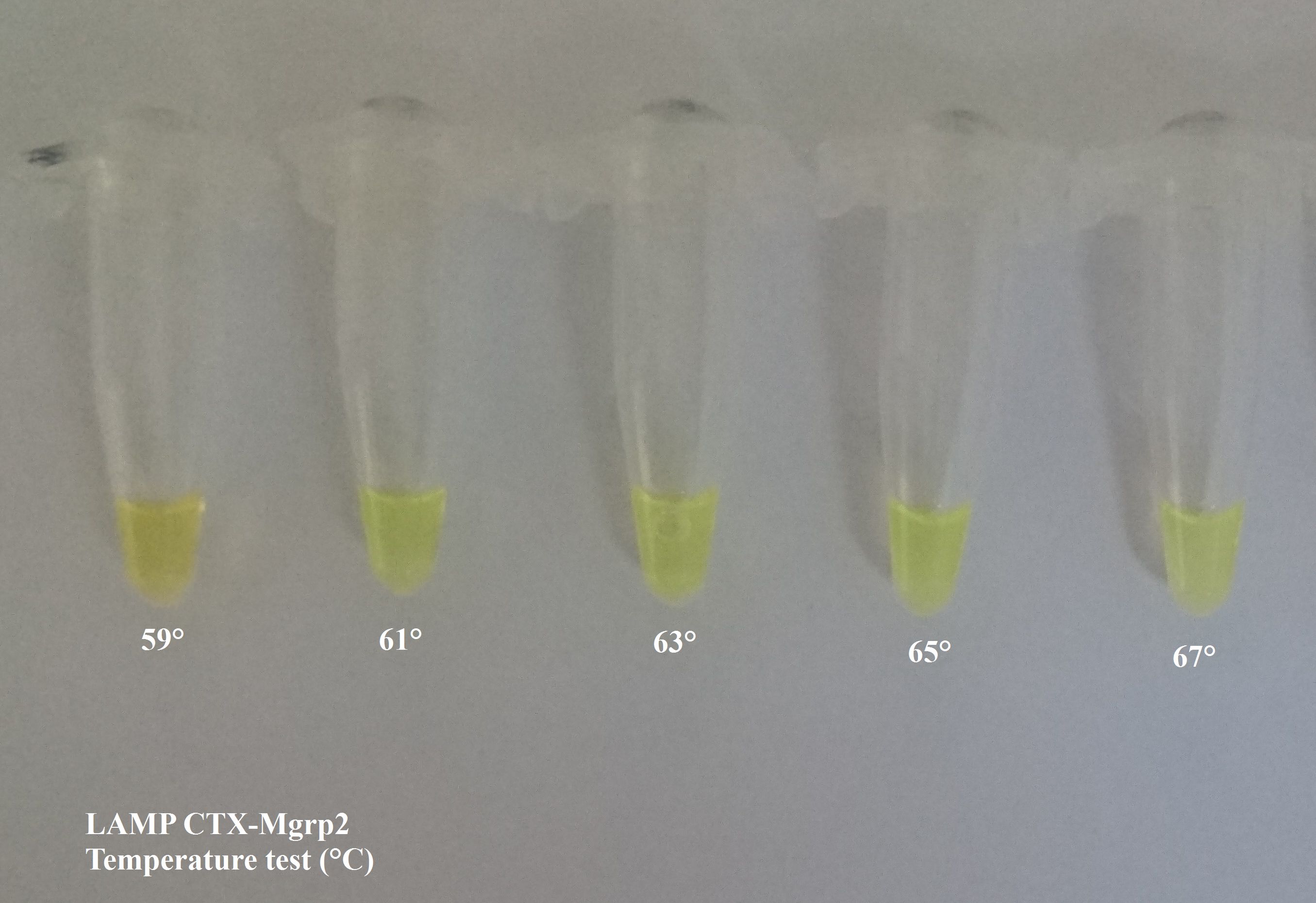

Supplement: S10 Fig — Betaine at 0.8M, MgSO4 at 7mM and amplification for 1H, the LAMP reaction was performed at 59, 61, 63, 65 and 67°C. The reaction products were visualized after coloration with the Sybr Green I dye. (JPG) [file pone.0200421.s010.jpg]

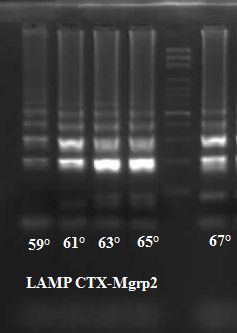

Supplement: S11 Fig — With 0.8M of betaine, MgSO4 at 7mM and amplification for 1H, the LAMP reaction was performed at 59, 61, 63, 65 and 67°C. The products were loaded onto a 2% agarose gel for analysis. (JPG) [file pone.0200421.s011.jpg]

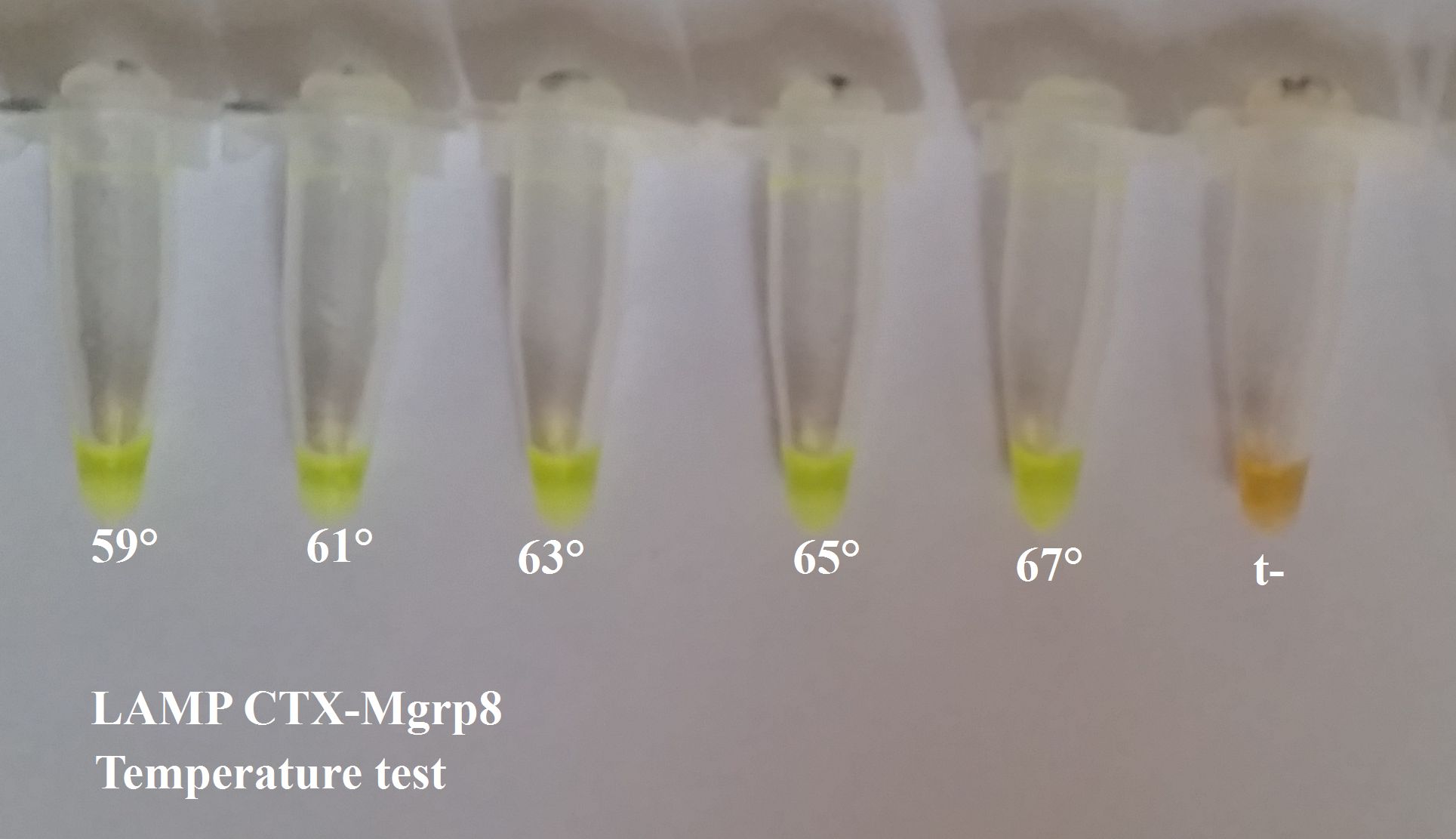

Supplement: S12 Fig — With 0.8M of betaine, MgSO4 at 7mM and amplification for 1H, the LAMP reaction was performed at 59, 61, 63, 65 and 67°C. The reaction products were visualized after coloration with the Sybr Green I dye. (JPG) [file pone.0200421.s012.jpg]

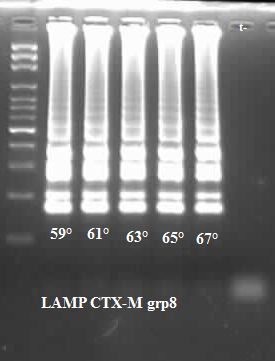

Supplement: S13 Fig — With 0.8M of betaine, MgSO4 at 7mM and amplification for 1H, the LAMP reaction was performed at 59, 61, 63, 65 and 67°C. The products were loaded onto a 2% gel for analysis. (JPG) [file pone.0200421.s013.jpg]

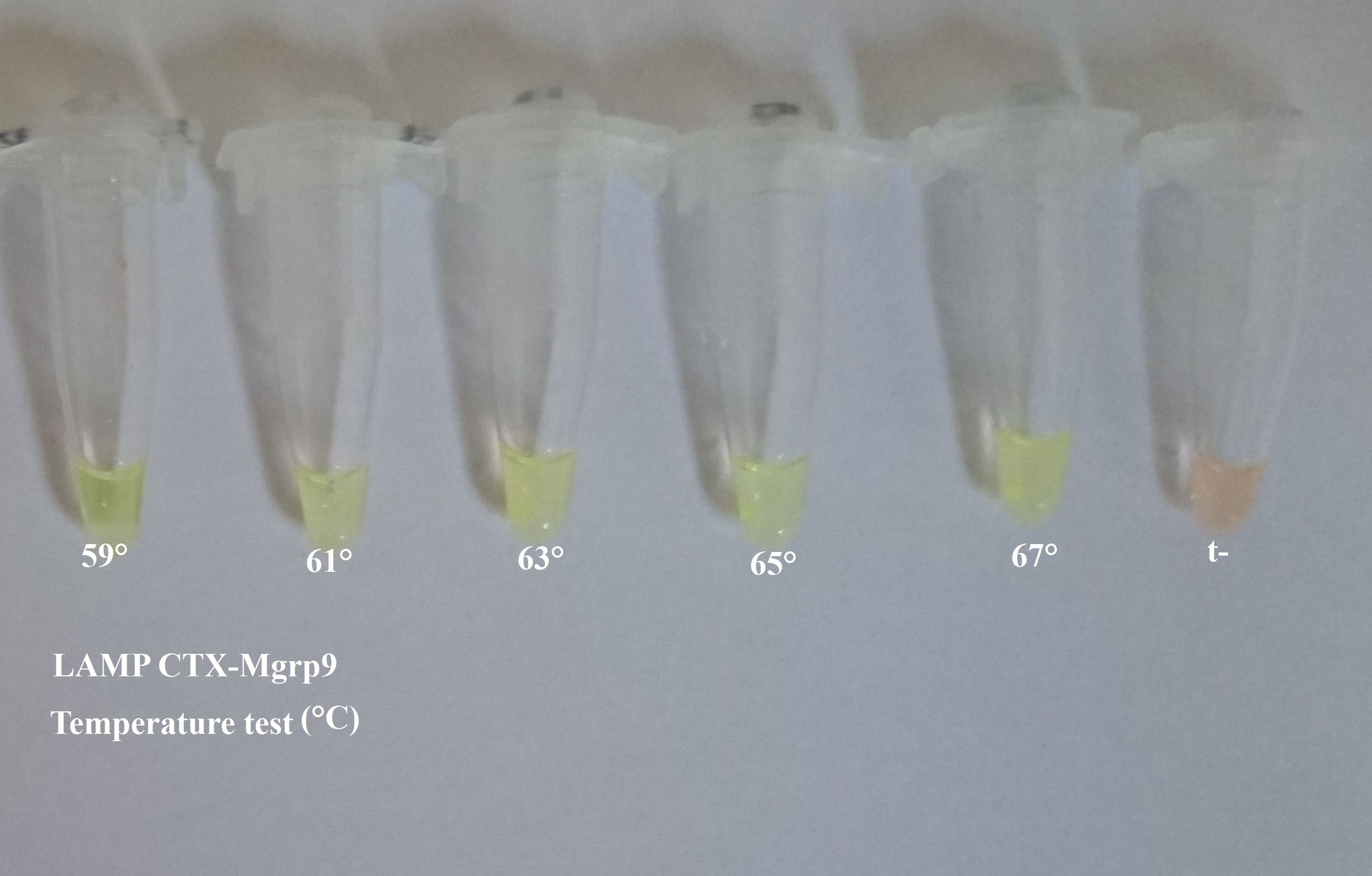

Supplement: S14 Fig — Betaine concentration maintained at 0.8M, MgSO4 at 7mM and amplification for 1H, the LAMP reaction was performed at 59, 61, 63, 65 and 67°C. The reaction products were visualized after coloration with the Sybr Green I dye. (JPG) [file pone.0200421.s014.jpg]

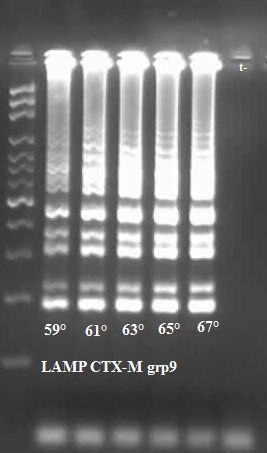

Supplement: S15 Fig — With 0.8M of betaine, MgSO4 at 7mM and amplification for 1H, the LAMP reaction was performed at 59, 61, 63, 65 and 67°C. The products were loaded onto a 2% gel for analysis. (JPG) [file pone.0200421.s015.jpg]

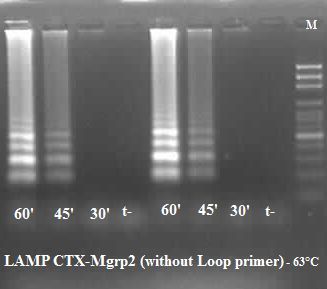

Supplement: S16 Fig — Betaine concentration maintained at 0.8M, MgSO4 at 7mM, amplification temperature at 63°C for 1H, the LAMP reaction was performed during 30, 45 and 60min. The reaction products were loaded onto a 2% agarose gel for analysis. (JPG) [file pone.0200421.s016.jpg]

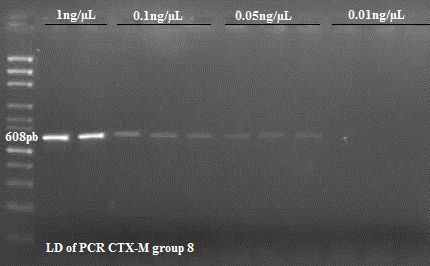

Supplement: S17 Fig — Various amount of template DNA (1, 0.1, 0.05 and 0.01ng/μl) were used. Conventional PCR products were analyzed on agarose gel. (JPG) [file pone.0200421.s017.jpg]

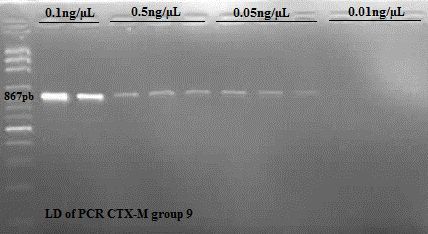

Supplement: S18 Fig — Various amount of template DNA (1, 0.1, 0.05 and 0.01ng/μl) were used. Conventional PCR products were analyzed on agarose gel. (JPG) [file pone.0200421.s018.jpg]
